# Expression and Purification of Hybrid LL-37Tα1 Peptide in *Pichia pastoris* and Evaluation of Its Immunomodulatory and Anti-inflammatory Activities by LPS Neutralization

**DOI:** 10.3389/fimmu.2019.01365

**Published:** 2019-06-14

**Authors:** Baseer Ahmad, Quratulain Hanif, Wei Xubiao, Zhang Lulu, Muhammad Shahid, Si Dayong, Zhang Rijun

**Affiliations:** ^1^State Key Laboratory of Animal Nutrition and Feed Sciences, College of Animal Science and Technology, China Agricultural University, Beijing, China; ^2^National Institute for Biotechnology and Genetic Engineering, Pakistan Institute of Engineering and Applied Sciences, Faisalabad, Pakistan; ^3^College of Veterinary Medicine, China Agricultural University, Beijing, China

**Keywords:** hybrid peptides, yeast expression, LPS neutralization, immunomodulatory, apoptosis

## Abstract

This study pertains to the new approach for the development of hybrid peptide LL-37Tα1 and its biomedical applications. A linear cationic hybrid peptide, LL-37Tα1 was derived from two parental peptides (LL-37 and Tα1) recognized as potent anti-endotoxin without any hemolytic or cytotoxic activity. We successfully cloned the gene of hybrid peptide LL-37Tα1 in PpICZαA vector and expressed in the *Pichia pastoris*. The recombinant peptide was purified by Ni-affinity column and reverse-phase high performance liquid chromatography (RP-HPLC) with an estimated molecular mass of 3.9 kDa as determined by SDS-PAGE and mass spectrometry. We analyzed the LPS neutralization by limulus amebocyte lysate (LAL) activity and the results indicate that the hybrid peptide LL-37Tα1 directly binds endotoxin and significantly (*p* < 0.05) neutralizes the effect of LPS in a dose-dependent manner. Lactate dehydrogenase (LDH) assay revealed that LL-37Tα1 successfully reduces the LPS-induced cytotoxicity in mouse RAW264.7 macrophages. Moreover, it significantly (*p* < 0.05) decreased the levels of nitric oxide, proinflammatory cytokines including TNF-α, IL-6, IL-1β, and diminished the number of apoptotic cells in LPS-stimulated mouse RAW264.7 macrophages. Our results suggest that the *P. pastoris* expression system is cost-effective for commercial production of the immunomodulatory and anti-inflammatory hybrid peptide (IAHP) LL-37Tα1 and the peptide may serve as effective anti-endotoxin/anti-inflammatory agent with minimal cytotoxicity.

## Introduction

Antimicrobial peptides (AMPs) are important components of the innate immune response against microbes. Besides plants and animals, AMPs have been identified in many other organisms including microorganisms (fungi, bacteria, archaea, algae, protozoa) (1–4). AMPs have secured additional attention because of their small size, amphipathic nature, and having potent activity against bacterial, viral, and fungal infections (5). AMPs that bind to LPS and neutralize its effect could have potential clinical applications (6, 7). The development of bacterial resistance toward conventional antibiotics has turned into a critical therapeutic emergency (8, 9). Antibiotics have been commonly used in the treatment of inflammation but they have many sides effects (10). Antibiotics can increase the release of bacterial LPS by killing bacteria and activate the immune system to secrete cytokines and produce lethal shock (11). LPS is an integral component of Gram-negative bacteria's outer membrane, that is released after lysis of bacteria and leads to a number of pathophysiological conditions like fever, leukopenia, intravascular coagulation, and sepsis (12, 13). Therefore, over the past decades, there has been a dire need for new anti-inflammatory peptides that have both antibacterial and LPS neutralizing activities (10).

LL-37 is an α-helical peptide discovered in 1995 in human leukocytes. It is comprised of 37-amino acid and attracts much research consideration (14). The LL-37 peptide plays a significant role as a primary line of defense against local and systemic infection of the microbes while reducing the inflammation. Besides antimicrobial activities, the LL-37 peptide also has the ability to enhance immunity and bind with the host cell surface molecules (11, 15). Additionally, LL-37 directly binds to LPS and neutralize its biological activity because it suppresses interleukin-6 (IL-6), interleukin 1β (IL-1β) and LPS-induced tumor necrosis factor-α (TNF-α) (16, 17). Henceforth, LL-37 is a favorable peptide for the control of inflammation and minimize the influence of endotoxin (18, 19).

Thymosin α1 (Tα1) is another peptide which originates from natural thymic peptide consisting of 28 amino acids (20) and is derived through cleavage from its precursor prothymosin α (proTα) (21). Tα1 has an important role to control infectious diseases and it regulates the immune response through a primary action on the cells of the innate immune system (22, 23). Tα1 has several immunomodulatory functions and it has been used in the treatment of immune dysfunctions i.e., hepatitis B and C, cancer, and sepsis (24). Importantly, it has been shown that Tα1 increases proteins expression on the surface of tumor cells and arbitrate antigen production such as major histocompatibility class (MHC) I, II, beta-2 microglobulin (25), and tumor-specific antigen (26, 27).

Hybridizing different AMPs is most effective method to obtained hybrid peptide having elevated antibacterial, anti-inflammatory, and less cytotoxic capabilities (28, 29). In the previous report from our laboratory, two hybrid peptides cecropin A (1-8)-LL37 (17-30) (30) and melittin (1-13)-LL37 (17-30) (31) were designed and expressed in *Escherichia coli (E.coli)* system. Antibacterial activities of both hybrid peptides were also investigated. Although, *E.coli* is a relatively easy expression system but its inability to fold the fusion proteins and lack of proper post-translational modification (i.e., glycosylation, proline cis/trans isomerization, lipidation, and sulphation) limit the expression of many proteins (32). Generally, this system is not suitable for proteins that contain high disulphide connectivity (33, 34). *E. coli* expressed protein also retain amino-terminal methionine, which affects protein stability (35). Whereas, *Picha pastoris* (*P. pastoris*) expression systems offer significant advantages including tightly regulated alcohol oxidase I promotor (AOX1) (36), a strong respiratory system that facilitates high cell densities (37–39), post-transcriptional modification and formation of disulfide bond. Another leading end benefit is the easy and convenient separation and purification of the target protein through the secretory yeast expression system (40). *P.pastoris* expression system has been extensively used to successful expression of many antimicrobial and anti-inflammatory hybrid peptides such as CA-MA (41), CecropinAD (42), and lunasin-4 (43).

In the present study, we hypothesized that the combination of LL-37 (24 amino acid) and Tα1 (8 amino acid) may have augmented LPS neutralization, its immunomodulatory, anti-inflammatory activity along with least cytotoxic effects. Therefore, we synthesized and expressed the hybrid peptide LL-37Tα1, in methylotrophic yeast expression system and investigated its bioactivities.

## Materials and Methods

### Reagents, Strains, Vectors

The Gel Extraction kit, Plasmid Mini kit, and DNA extraction kit for yeast were procured from Tiangen Biotech (Beijing, China). The restriction enzyme *Kpn I, Xba I*, and *Sac I* were obtained from TaKaRa Biotech Inc. (Dalian, China). PCR reagents, were sourced from Tiangen Biotech (Beijing, China). Zeocin™ was purchased from Invitrogen (Carlsbad, CA, USA). *Pichia pastoris* (strain X-33), *E. coli* (strain *DH5*α), expression vectors pPICZαA (Invitrogen, USA), protein markers (Thermo Fisher Scientific, USA) and *E.coli* LPS (O55: B5, Sigma-Aldrich, USA) were routinely available in our laboratory.

### Construction of Expression Plasmid pPICZαA-LL-37Tα1

The hybrid LL-37Tα1 peptide was optimized and rare codons were removed according to the amino acid sequence by using the JAVA codon adaptation tool (JCAT) http://www.jcat.de/Start.jsp). The sequence coding LL-37Tα1 has combined accordingly as per favored codon usage of *P. pastoris* by Tsingke Biological Technology Co, Ltd Beijing. The 129 bp fragment of LL-37Tα1 was implanted at the restriction site *Kpn I* and *Xba I* with 6 × Histidine tag (6 × His-tag) of the expression vector pPICZαA and transformed to *E.coli (Dh*α*5)*. Positive transformants were then screened on Luria Bertani (LB) plates containing yeast extract (5 g/L), tryptone (10 g/L), and NaCl (10 g/L) and confirmed by PCR using primers as Sense; 5′-TCGGTAAGGAATTCAAGAGA-3′ Antisense 5′-GATGATGTTCAACAACTTCC-3′ and sequencing was carried out (Tsingke Biotech). PCR conditions were followed as 35 cycles (95°C/40 s; 55°C/40 s; 72°C/50 s) and final extension at 72°C/10 min. The positive recombinant plasmid (pPICZαA-LL-37Tα1) was extracted by Tianprep plasmid kit (Tiangen, Beijing, China).

### Transformation and Expression of Hybrid LL-37Tα1 Peptide

The expression plasmid (pPICZαA-LL-37Tα1) was linearized with *Sca I* and transformed into *P. pastoris* (X-33) by electroporation according to manufacturer's directions (Invitrogen, USA). All zeocin resistant colonies in yeast extract peptone dextrose (YPDS) medium (1% yeast extract, 2% peptone, 2% dextrose, IM sorbitol, 2% agar, and zeocin 100 μg/ml) were screened. The positive strains were verified by PCR using 5′ alcohol oxidase 1 (AOX1) and 3′ AOX1 promoter region primers and subsequent sequencing (Tsingke Biotech). The positive recombinant (PpICZαA-LL-37Tα1) yeast cells were cultured for 22 h in a shaker containing 50 ml buffered glycerol complex (BMGY) medium (1% yeast extract, 2% peptone, 1.34% YNB, biotin 4 ×10^−5^%, 1% glycerol and 100 mM potassium phosphate, pH 6.0) to OD_600_ = 5.0. The yeast cells were collected by centrifugation at 2,000 × g for 5 min. At the room temperature, the cells were resuspended in buffered methanol-complex (BMMY) medium (1% yeast extract, 2% peptone, 1.34% YNB, 4 ×10^−5^ % biotin, 1% methanol and 100 mM potassium phosphate, pH 5.0). 1% methanol was added every 24 h and culture supernatant was analyzed for expression of a hybrid peptide by Tricine-sodium dodecyl sulfate-polyacrylamide gel electrophoresis (Tricine-SDS-PAGE) till optimization was obtained at 144 h. Protein concentration was detected by using the Bio-Red dye agent with bovine serum albumin (BSA) as a standard (44).

### Purification and Analysis of Hybrid LL-37Tα1 Peptide

Purification of the recombinant LL-37Tα1 peptide was done according to the procedure described hitherto (45) with slight modification. After 144 h of 1% methanol induction, the culture supernatant was collected by centrifuge at 12,000 × g for 20 min. The supernatant comprising LL-37Tα1 peptide attached with 6 × His-tag was filtered and loaded to 1 ml His Trap Chelating Ni-affinity column (Bio-Beads TM, Sweden). The column was equilibrated with 1 × phosphate buffer (PB) and 10 mM imidazole. The recombinant peptide was eluted by using a different concentration linear gradient of imidazole (50–500 mM). The eluted proteins were analyzed by Tricine- SDS-PAGE and the protein concentration was determined by using the Bio-Red reagent with BSA standard (44).

After initial purification, the recombinant LL-37Tα1 peptide was further purified by using reversed phase-high performance liquid chromatographic method (RP-HPLC) comprised of C18 column (4.6 ×250 mm, 5 μm) with a linear gradient of acetonitrile (0–100% for 30 min) containing 0.1% Trifluoroacetic acid (TFA) at a flow rate of 1.0 ml/min. The elution peaks of recombinant peptide were monitored at 220 nm. The activity of the fraction containing recombinant LL-37Tα1 peptide was collected and verified by using LPS neutralization assay (46). The purified hybrid LL-37Tα1 peptide was diluted with milli-Q water and filtered through 0.22 μm and passed through electrospray ionization mass spectrometry ESI-MS/MS.

### Activity Assay of Recombinant LL-37Tα1 Peptide

#### Determination of LPS Neutralization

The neutralization of endotoxin by the parental (LL-37) and hybrid peptide (LL-37Tα1) was evaluated using a Chromogenic limulus amebocyte lysate (LAL) assay according to manufacturer's instructions with some modification. A constant deliberation of endotoxin (1 EU/ml) was incubated with a varied concentration of the parental and hybrid peptide (0 to 50 μg/ml) in the wells of a pyrogenic sterile microtiter plate at 37°C. The 50 μL aliquots concentrate of LAL reagent was added and incubated for 10 min at 37°C. On the addition of 100 μL chromogenic substrate, yellow color appeared. The reaction was stopped by adding 25% HCl and the absorbance was measured at 545 nm (46).

#### Hemolytic Activity

Heparinized mouse red blood cells (RBCs) were used to determine the hemolytic activity of the parental (LL-37) and hybrid peptide (LL-37Tα1) with slight modification (47, 48). Fresh mouse RBCs (4 mL) were collected at 1,500 rpm for 10 min at 4°C. The cells were diluted to 10% hematocrit after subsequent washing with phosphate buffer saline. After that cells were incubated with the parental and hybrid peptide with concentrations ranging from 10 to 50 μl for 1 h at 37°C, then centrifuged at 3,500 rpm for 5 min. The absorbance of supernatant was measured at 414 nm. PBS and Triton X-100 were used as negative and positive controls, respectively.

#### Cell Culture

The mouse RAW 264.7 macrophages were cultured in Dulbecco's modified eagle's medium (DMEM) supplemented with antibiotics (100 μg/ml streptomycin and 100 U/ml penicillin) and 10% fetal bovine serum in a humid, 5% CO_2_ chamber at 37°C.

#### Lactate Dehydrogenase Activity (LDH) Assay

LDH assay was employed to evaluate the cytotoxic effect of LL-37Tα1 peptide on mouse RAW264.7 macrophages treated with or without LPS as defined previously with slight modification (49, 50). LPS (1 μg/ml) treated and untreated cells (1 ×10^5^ cells/mL) were exposed to LL-37Tα1 (10 to 50 μg/ml), while only LPS treated cells were exposed with parental peptide to check the comparative effect with hybrid peptide and incubated for 24 h. The cell supernatants were collected and LDH activity was measured according to the LDH assay kit's instruction (Dojingdo Laboratories, Kumamoto, Japan).

#### Inhibition of Nitric Oxide (NO) Production in LPS-induced Mouse RAW264.7 Macrophages

LPS (1 μg/ml) treated mouse RAW264.7 macrophages were incubated with various concentration of parental LL-37 and hybrid LL-37Tα1 peptide (20–50 μg/ml). After incubation of 24 h, the culture supernatant was collected and NO assay was performed. The same volume of Griess reagent (1% sulfanilamide in 5% phosphoric acid and 0.1% naphthylethylene diamine dihydrochloride) was interspersed with 100 μL of the culture medium and further incubated for 15 min (47). The absorbance of the sample was measured at 550 nm.

#### Determination of Pro-inflammatory Cytokines in LPS-induced Mouse RAW264.7 Macrophages

The levels of TNFα, IL-6, and IL-1β in the culture supernatant were assessed after the addition of LPS (1 μg/ml) to mouse RAW264.7 macrophages (5 ×10^5^ cells /well) in the absence and presence of LL-37 and LL-37Tα1 peptide (10–50 μg/ml). The cells were incubated for 24 h at 37°C with 5% CO_2_. The secretion of pro-inflammatory cytokines was measured in the culture supernatants by using mouse cloud-clone corp protein antibodies enzyme-linked immunosorbet assay (ELISA) kit (Houston, USA). The levels were quantified and analyzed at 450 nm absorbance.

#### Flow Cytometry

LPS-stimulated apoptosis in mouse RAW264.7 macrophages was determined by using (Propidium iodide) PI and annexin (V conjugated to green-fluorescent FITC dye) V-FITC staining method. The mouse RAW264.7 macrophages were cultured as described above and challenged with LPS (10 μg/ml) with the absence and presence of the LL-37 and LL-37Tα1 peptide for 4, 12, and 24 h. The cells were washed with ice-cold PBS three times and stained with FITC annexin V and PI following apoptosis detection kit (Becton Dickinson Fraklin Lakes, NJ, USA) as described earlier (48). Apoptosis rate was articulated by using FACSA via flow cytometry (Becton Dickinson, USA).

### Statistical Analysis

The data were shown as mean ± standard deviation (SD) of the three independent experiments. The data were statistical evaluated by the one-way analysis of variance (ANOVA) followed by least significant difference (LSD) test for multiple comparisons, and *post hoc* test using SPSS 19.0 (SPSS, Inc., Chicago, IL, USA). Moreover, *p* < 0.05 and *p* < 0.01 represent the significance and highly significance differences.

## Results

### Construction and Expression of pPICZαA-LL-37Tα1

**Figure 1:**
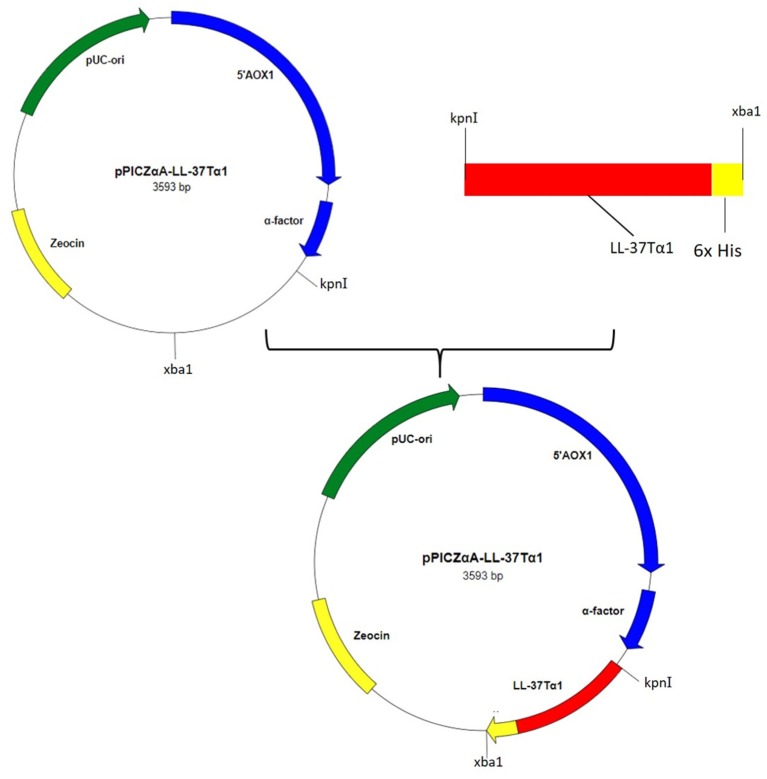
Construction of recombinant plasmid pPICZαA-LL-37Tα1.

**Figure 2:**
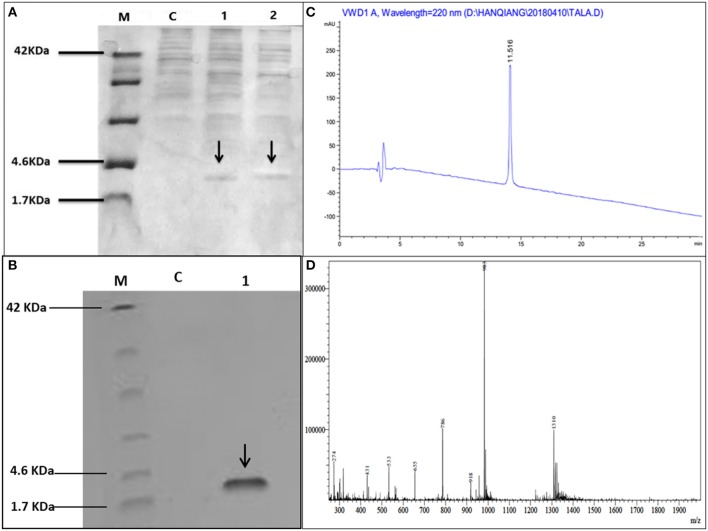
Tricine-SDS-PAGE and analysis of recombinant peptide, **(A)** Tricine-SDS-PAGE of the cell culture media from *P.pastoris* expressing secreted LL-37Tα1. Lane M, molecular weight markers; Lane C, control (supernatant of X33/PpICZαA); Lane 1 and 2 (supernatant X33/PpICZαA-LL-37Tα1) peptide expression after methanol (144 h) induction and arrow in the lane indicated 3.9 kDa polypeptide. **(B)** Tricine-SDS-PAGE of recombinant purified LL-37Tα1. Lane M, molecular weight markers; Lane C, control (X33/PpICZαA); Lane 1, a sample of the purified recombinant peptide and arrow indicated LL-37Tα1 (3.9 kDa) expression. **(C)** The elution pattern of RP-HPLC C18 column of the purified recombinant LL-37Tα1 and the high peak indicates fraction that contains LL-37Tα1. **(D)** ESI-MS analysis of purified recombinant LL-37Tα1 peptide.

Hybrid peptide gene was amplified through PCR and 129 bp DNA fragment encoding a C-terminal 6 × His tag which was attached with *Kpn I* and *Xba I* restriction enzyme site at it's 5′ and 3′ end, respectively, was inserted into pUC57 vector. This fragment was double-digested with restriction enzymes (*Kpn I* and *Xba I*) and was a frame clone to attach to the 3′ end of the α-factor secretion signal, downstream AOX1 promoter of the expression plasmid pPICZαA to result in a recombinant vector named pPICZαA-LL-37Tα1. The insertion was then verified by restriction enzyme digestion analysis and sequencing. The construction procedure of pPICZαA-LL-37Tα1 is shown in (Figure 1). The expression vector pPICZαA-LL-37Tα1 was treated with *Sac I* and linearized fragment was later transformed into *P. pastoris* X-33 competent cells by electroporation. The ten zeocin (100 μg/ml) resistant colonies were selected and confirmed by LL-37Tα1 and PpICZAαA specific primers by PCR. Our result revealed that target pPICZαA-LL-37Tα1 sequence successfully integrated into the host cells. The positive transformed colonies were then induced by 1% pure methanol (v/v) at 144 h after optimization of methanol concentration. After induction of methanol, the culture supernatant was collected and analyzed by Tricine-SDS-PAGE and silver staining. As expected, the 3.9 kDa molecular weight recombinant hybrid LL-37Tα1 peptide was observed (Figure 2A).

### Purification, RP-HPLC, and Mass Spectrometry Analysis of Hybrid LL-37Tα1 Peptide

The LL-37Tα1 peptide was purified by Ni-NTA affinity chromatography column after centrifugation of culture medium. The pure hybrid peptide was eluted with 400 and 500 mM imidazole. As shown in (Figure 2B), SDS-PAGE of the purified peptide indicated single band conforming to the estimated 3.9 kDa size. (Figure 2C), which yielded 5 mg of pure recombinant peptide. After purification, the hybrid peptide was diluted in milli-Q water and filtered through a 0.22 μM filter and then adjusted to ESI-MS/MS. Mass spectrometry of the purified LL-37Tα1 displayed a single non-dispersed signal (Figure 2D). The average mass of the molecular ion [M+5H^+^]^5+^ was 786 Da, [M+4H^+^]^4+^ 983 Da, and [M+3H^+^]^3+^ 1310 Da which corresponds to the molecular mass of 3927 Da for the recombinant LL-37Tα1. This result indicated that recombinant peptide was removed from the N-terminus successfully.

### Immunomodulatory and Anti-inflammatory Response of Hybrid LL-37Tα1 Peptide

#### Recombinant LL-37Tα1 Neutralize LPS

**Figure 3:**
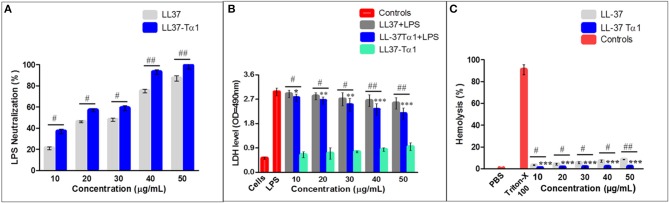
LPS neutralization, cytotoxicity, and hemolytic activity of parental and recombinant LL-37Tα1 peptide. **(A)** LPS neutralization by LL-37 and LL-37Tα1 determined using an endotoxin quantitation kit. Mean values presented; *n* = 3 ± SD (^#^*p* < 0.05 and ^##^*p* < 0.01 showed comparison of LL-37 vs. LL-37Tα1). **(B)** LL-37αT1 peptide decreased cytotoxicity in the cultured medium of LPS-infected mouse RAW264.7 macrophages. Data represented as mean ± standard deviation (SDs) of independent experiments. ^*^p < 0.05,^**^p < 0.01,^***^p < 0.001, vs. LPS. Whereas, ^#^*p* < 0.05 and ^##^*p* < 0.01 indicates significant difference compared with parental LL-37 peptide. **(C)** Hemolytic activities of LL-37Tα1 against mouse RBCs. The data correspond to the mean values of three independent experiments and are expressed as a percentage of hemolysis ± standard deviation (^***^p < 0.001 vs. Triton X-100). While, ^#^*p* < 0.05 and ^##^*p* < 0.01 indicates significant difference compared with parental LL-37 peptide.

Under the physiological conditions, The LL-37Tα1 peptide has a net charge of +7 and we predicted that it would bind and neutralizes the LPS. LAL test is an extremely sensitive indicator of the presence of free non-neutralized LPS as described previously (51–53), we determined the capability of the parental and hybrid peptide to neutralize LPS *in vitro* by using this test. Our result exhibited that LL-37 (40 and 50 μg/ml) was capable of neutralizing LPS (75.241% ±2.100, 87.361% ±3.210, respectively) and LL-37Tα1 (93.231% ± 2.3828, 99.131% ± 3.284, respectively) in a dose-dependent manner Whereas, hybrid peptide significantly increased the neutralization of LPS in comparison to parental peptide (Figure 3A).

#### Cytotoxicity and Hemolytic Activity of Recombinant LL-37Tα1 Peptide

The toxicity of the hybrid peptide was determined by using LDH assay with and without LPS in mouse RAW 264.7 macrophages. Data indicate that LPS infection in the absence of hybrid peptide induced higher release of LDH at 24 h (3.2 ± 0.051) as compared to the combined treatment of LPS and LL-37Tα1 peptide (10 to 50 μg/ml). This indicates that LPS damaged the mouse RAW264.7 macrophage's cell membrane but LL-37Tα1 peptide significantly (*p* < 0.001) neutralized the LPS and decreased the LDH level (2.38 ± 0.066) at 40 μg/ml and (2.16 ± 0.037) at 50 μg/ml, respectively (Figure 3B). Furthermore, the recombinant hybrid peptide also reduced LPS-induced cytotoxicity as compared with parental peptide. The hemolytic activity of hybrid peptide was examined by lysing mouse RBCs (Figure 3C). As compared with the control group 0% (*p* < 0.001) hemolysis was observed in the treated cells and hybrid peptide caused significantly less hemolysis than LL-37. Altogether, these findings provide evidence that the hybrid LL-37Tα1 peptide does not have cytotoxic and hemolytic properties.

#### LL-37Tα1 Down Regulates LPS-stimulated Inflammatory Response in Mouse RAW264.7 Macrophages

**Figure 4:**
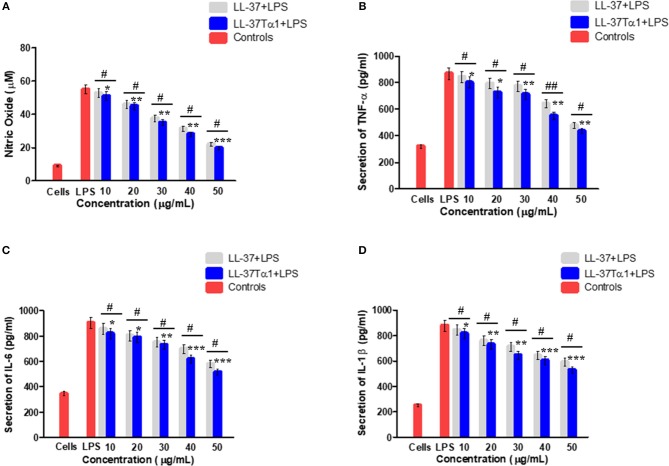
Effect of LL-37 and recombinant LL-37Tα1 peptide on LPS-induced inflammatory response in mouse RAW264.7 macrophages. **(A)** Nitric oxide (NO) production, **(B)** level of TNF-α, **(C)** IL-6, and **(D)** IL-1β, Cell were infected by LPS to stimulate inflammation and treated with various concentration of LL-37 and LL-37Tα1. After 24 h incubation, culture media were collected and inflammatory cytokines level were determined. Values are means ± SD of three independent experiments. ^*^p < 0.05, ^**^p < 0.01, ^***^p < 0.001, vs. LPS. While, ^#^*p* < 0.05 and ^##^*p* < 0.01 indicates significant difference compared with parental LL-37 peptide.

The ability of LL-37Tα1 to neutralize LPS prompted us that it could suppress the inflammatory response induced by LPS. To address this query, we appraised the effect of hybrid LL-37Tα1 peptide on LPS-induced secretion of NO and the proinflammatory cytokines including TNF-α, IL-6, and IL-1β in mouse RAW264.7 macrophages. Our results suggested that LPS significantly increased the NO level from mouse RAW264.7 cells as compared to control group (55 vs. 9 μM), and this NO level was reduced (33 and 28 μM) after treatment with LL-37Tα1(40–50 μg/ml) as shown in (Figure 4A). Similarly, the analysis of TNF-α, IL-6, and IL-1β by ELISA to depict that LL-37Tα1 significantly decreased the secretion of TNF-α, IL-6 and IL-1β in mouse RAW264.7 macrophages (868.93, 903.88, and 878.69 pg/ml, respectively) as compared with secretion in cells infected with LPS only (Figures 4B–D). LL-37Tα1 treatment (40 and 50 μg/ml) significantly (*p* < 0.001) reduced TNF-α concentration550.93 pg/ml and 437.41 pg/ml, respectively (Figure 4B), IL-6 reduced 615.73 pg/ml, 517.25 pg/ml (Figure 4C), and IL-1β showed a reduction of 601, 527 pg/ml, respectively (Figure 4D). The results of the current study reveal that our hybrid peptide LL-37Tα1 has significant anti-inflammatory activity. Moreover, the hybrid peptide LL-37Tα1 exhibited more anti-inflammatory activities as compared to parental peptide LL-37 (Figures 4A–D).

#### LPS-stimulated Apoptosis in Mouse RAW264.7 Macrophages

**Figure 5:**
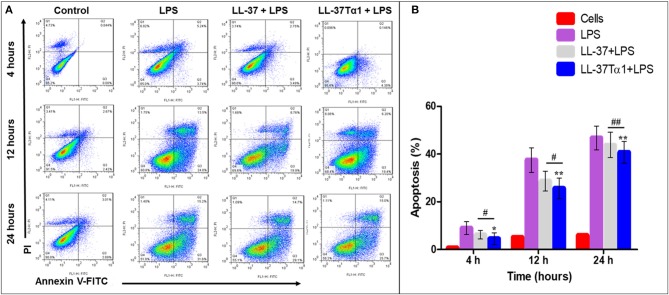
Apoptosis of mouse RAW264.7 macrophages treated with LPS alone and co-infection with parental and our hybrid peptide. Mouse RAW264.7 macrophages were treated with LPS and without LL-37 and LL-37Tα1+LPS at 4, 12, and 24 h with FITC-conjugated annexin-V (Green) and PI (Red). **(A)** The stained mouse RAW264.7 macrophages were examined by flow cytometry, Control represents normal cells, the middle panel LPS representative mouse RAW 264.7 macrophages treated with LPS only, and right panel LL-37+LPS, LPS+LL-37Tα1 reveals mouse RAW64.7 macrophages treated with LPS and peptide. **(B)** Percentile value of apoptotic cells after treatment of LPS alone or with a hybrid peptide with mouse RAW64.7 macrophages. ^*^p < 0.05 and ^**^p < 0.01 vs. LPS indicate the significance and highly significance difference. ^#^*p* < 0.05 and ^##^*p* < 0.01 indicates significant difference in hybrid peptide compared with parental LL-37 peptide.

To investigate the effect of LL-37 and LL-37Tα1 peptide on LPS-induced apoptosis, mouse RAW264.7 macrophages were cultured and infected with LPS alone and with LPS plus parental and hybrid peptides for 4, 12, and 24 h. Then mouse RAW264.7 cells were stained with annexin V-FITC and PI as per manufacturer's instructions and analyzed by flow cytometry. As shown in Figure 5A, LPS increased the number of both early and late apoptotic cells at 4, 12, 24 h as compared with control and treated group. However, LL-37Tα1 and LPS combined treatment significantly reduced (*p* < 0.01) the number of apoptotic cells as compared to both LPS and LL-37 plus LPS group (Figure 5B). Our results depicted that our designed peptide neutralizes LPS and ultimately reduces apoptosis in mouse RAW264.7 macrophages.

## Discussion

The emergence of microbial resistance to antibiotics has become a major issue worldwide. AMPs are new and effective antibacterial agents as they serve as a natural defense against harmful pathogens of vertebrates (54, 55). AMPs are deliberated one of the rare preferences to use as an alternative or in combination with traditional antibiotics that tend to lead a variety of resistant bacteria. AMPs have a positive net charge that permits them to interrelate with bacterial membrane and LPS which have negative charge (56, 57). The binding with LPS not only stimulates the mechanism by which AMPs kill bacteria but also neutralize LPS in certain cases. Therefore, AMPs can be considered instantaneously antimicrobial and anti-inflammatory candidate drugs for the diseases (56). In the recent years, the scientists have endeavored to modify the amino acid sequence of the parental peptides in order to improve the expression and obtain the best bacteriostatic, immunomodulatory, and anti-inflammatory activities. Furthermore, the conserved amino acid sequence of the peptides have great impact on the above stated activities and also the proper replacement of some conserved sequence does not affect its activity but some suitable substitutions could improve the response of hybrid peptides (58). Hybridizing dissimilar parental peptides is an effective method to enhance the antibacterial and anti-inflammatory activities with minimum adverse effects (31, 59) such as cecropin, cathelicidin, magainin II, LL-37, and melititin (41, 60, 61).

There are different methods for the production of hybrid peptides i.e., extraction from natural resources, chemical synthesis, and recombinant expression (62). Due to high cost of chemical peptide synthesis, the methylotrophic yeast expression system provides an opportunity for the production of hybrid peptides in large aggregates (63). In the present study, hybrid peptide LL-37Tα1 was successfully expressed in *P. pastoris* in optimized expression conditions such as temperature, pH, and methanol induction. Comparatively, *P. pastoris* system is more operative in promoting disulfide bonding than bacterial expression system (64), which would be expected important for the activation of disulfide-containing LL-37Tα1 and recombinant peptide expression and secretion into the medium. The *P. pastoris* system has successfully expressed heterogenous peptides at high yields (65) while AOX1 promoter is responsible for transcription and alcohol oxidase activity in cells (66, 67). In the present study, After 144 h methanol (1%) induction, we obtained 40 mg/L peptide in the culture medium and our SDS-PAGE results showed that the size of recombinant LL-37Tα1 peptide is 3.9 kDa. The expression yield is higher than previously reported such as T-catesbeianin-1 (62), ceropinAD (42), and CA-MA (41). Moreover, the recombinant peptide was purified for functional and structural studies. For purification, we used the Ni-NTA affinity chromatography column (45) and RP-HPLC method as described earlier (42) and identified on SDS-gel profile. After two steps of purification, the 5 mg pure peptide was obtained from 200 ml medium. The result of ESI-MS analysis of pure peptide showed calculated molecular weight of 3927 Da.

In the present study, purified LL-37Tα1 peptide was subjected to LPS neutralization, cytotoxicity, and hemolytic activity assay, considered as important features of a hybrid peptide to be used as proficient antibiotic. LPS to constitute of three parts, lipid A, O-antigen and polysaccharide core. Lipid A is part of endotoxin that is responsible for the activation of LAL reagent (68, 69). In the present study, LAL test to depict that LL-37Tα1 peptide utilizes its antiendotoxin activity by binding lipid A portion of LPS and consequently blocking the biological effect of endotoxin.

Furthermore, the parental antimicrobial peptides have effective activities against both Gram-positive and negative bacteria but also reveal cytotoxic and hemolytic effect toward mammalian cells (68, 70). Our peptide efficiently neutralized LPS that comprises of a N-terminal region along with polar amino acids (net charge +7) and a C-terminal region with reduced hydrophobic amino acids as reported in previous study (71–73). It supported our hypothesis that this combination conferred strong anti-inflammatory activity with minimal cytotoxicity and significantly increased the LPS binding affinity these findings are in line with other studies (53, 62, 72, 74, 75). These features are assumed to reflect the robust electrostatic interaction between LL-37Tα1 peptide and LPS. Besides, LL-37Tα1 peptide showed more potent endotoxin neutralizing activity than that of parent peptides without cytotoxic and hemolytic activity.

In the current study, we also observed LPS-stimulated production of NO, TNF-α, IL-6, IL-1β in mouse RAW264.7 macrophages. Endotoxin is released when Gram-negative bacteria are killed or multiply in the host (76, 77) and induce inflammation, septic shock and sepsis (77, 78). As in inflammation, macrophages are activated and secrete NO at sites of injury to heal the damaged tissues and eliminate the cause (79). However, the over secretion of NO leads to variable inflammatory responses (80). Therefore, reducing the production of NO could be a new strategy against inflammatory disorders. The stimulated macrophages also produce a large number of proinflammatory cytokines which are involved in up-regulation of inflammation and might cause diseases such as hemorrhagic shock, multiple sclerosis, rheumatoid arthritis, ulcerative colitis, and atherosclerosis (81). Consequently, minimizing proinflammatory response is important to reduce inflammatory diseases. As compared to recombinant protein SPHF1 (82) and lunasin-4 (43), we found that LL-37Tα1 more efficiently inhibited the production of proinflammatory cytokines.

Previously, it has been reported that cationic peptides bind to the cell surface CD14 receptor on RAW264.7 macrophages and inhibit the LPS binding to the CD14 cell (17, 83–85). The finding of the present study to depict that hybrid LL-37Tα1 peptide suppressed cytokines expression induced by LPS in RAW264.7 macrophages. These features contemplate that LL-37Tα1 peptide is an attractive drug candidate for the treatment of endotoxin shock caused by Gram-negative bacterial infection.

Furthermore, LPS up-regulates adhesion molecules (86) coagulation factors (87, 88), and induces apoptosis (89, 90). In the case of the septic syndrome, macrophages play a major role to identify harmful components like LPS and release proinflammatory cytokines. Subsequently, apoptosis of macrophages leads to shock (91). In this regard, therapeutic approaches reducing macrophages activity are considered as useful tool against severe inflammatory conditions. In the present study, we investigated the effect of LL-37Tα1 on the LPS-induced apoptosis in mouse RAW264.7 macrophages by flow cytometry. Our findings specified that LL-37Tα1 hybrid peptide neutralizes LPS and reduces both early and late apoptosis as compared with LPS infected cells. Overall, these observations indicate that LL-37Tα1 is a auspicious peptide that could be developed for application in the medicine industry.

## Conclusions

**Figure 6:**
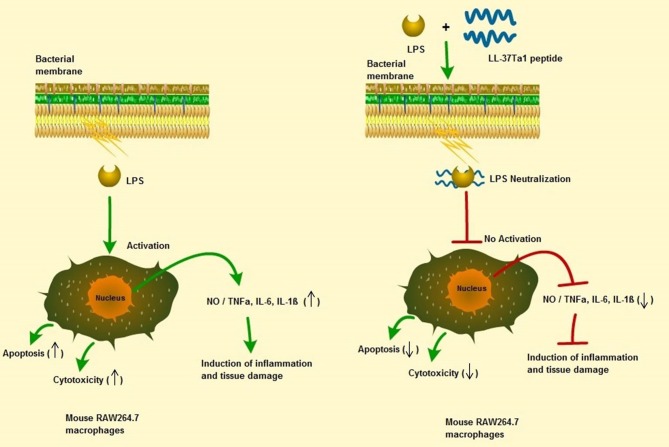
Schematic diagram of the potential mechanism by which LL-37Tα1 suppresses LPS-induced inflammatory responses in mouse RAW264.7 macrophages. Left, LPS alone; right LPS plus LL-37Tα1. The LL-37Tα1 binds with LPS and neutralizing it. (↑), activation; (**⊣**), no activation; (↑), upper regulate responses; (↓), lower regulate responses.

In this study, we successfully expressed and purified the hybrid peptide LL-37Tα1 in the *P. pastoris* system with expression vector PpICZαA. The recombinant peptide showed anti-endotoxin, immunomodulatory, and anti-inflammatory activities by binding with *E. coli* LPS. It inhibits NO and proinflammatory cytokines and reducing the number of apoptotic cells in LPS-induced mouse RAW264.7 macrophages with reduced cytotoxic and hemolytic activities, as graphical modeled in Figure 6. These results provide a strategy for recombinant production of hybrid LL-37Tα1 and also suggests that LL-37Tα1 could be a potential therapeutic agent for infectious diseases.

## Data Availability

This manuscript contains previously unpublished data. The name of the repository and accession number are not available.

## Author Contributions

BA wrote the paper. BA and QH performed the experiments. BA, QH, ZL, and ZR conceived and designed experiments. BA, QH, WX, and MS analyzed the data. ZR and SD guided the experiments. All authors helped to prepare the paper and approved the final version.

### Conflict of Interest Statement

The authors declare that the research was conducted in the absence of any commercial or financial relationships that could be construed as a potential conflict of interest.
